# Editorial: Trends in applications and improved production of biologically active metabolites using microbial fermentations

**DOI:** 10.3389/fmicb.2022.1065888

**Published:** 2022-11-22

**Authors:** Niranjan Koirala, Ameer Khusro, Sailesh Malla

**Affiliations:** ^1^Gandaki Province Academy of Science and Technology, Pokhara, Nepal; ^2^Department of Biotechnology, Centre for Research and Development, Hindustan College of Arts and Science, Chennai, India; ^3^Department of Strain Development, Discovery R&D, Chr. Hansen, Hørsholm, Denmark

**Keywords:** fermentation, microorganisms, genome mining, metabolomics, biologically active metabolites, chemotherapeutics

Microorganisms are considered factories of metabolites. Fermentation is one of the oldest processes of biotechnology which represents the production of bioactive metabolites from distinct groups of microorganisms (Koirala et al., 2014; Khusro and Aarti, 2022). A plethora of microbes from soil, marine, fermented foods, and fecal and other environmental sources produces diversified classes of bioactive metabolites *via* fermentation process.

Amino acids, nucleotides, and fermentation end products (ethanol and organic acids) are the primary metabolites which help in the growth and metabolism of microorganisms. On the other hand, secondary metabolites viz. enzymes, proteins, bacteriocins, antibiotics, pigments, growth hormones, etc. are generally synthesized during the stationary phase of microbial growth and exhibit disparate applications in nutrition, medicine, agriculture, food industries, environment, and livestock sectors (Koirala et al., 2017; Singh et al., 2017; Yang et al.). The production or secretion of these metabolites from microbes can be enhanced by optimizing independent variables *via* solid state or submerged fermentation process using cost-effective substrates (Martǎu et al., 2021).

Although a plethora of reports has been published in the past revealing the pivotal roles of microorganisms as bioactive metabolites producers, our understanding on the mechanism of production, its enhancement strategies, and its applications in diversiform sectors is still limited. The Research Topic entitled “*Trends in applications and improved production of biologically active metabolites using microbial fermentations*” is a significant attempt to bring together renowned scientists and researchers worldwide and spotlight the novel research activities being carried out in the theme of “biologically active metabolites from microbes.” This Research Topic comprises 11 articles covering crucial aspects of microbial fermentation and its associated metabolites' applications.

To facilitate the use of woody plant as a natural biomass resource for addressing the shortage of feed for ruminants in the tropics, Du et al. used PacBio SMRT sequencing method to explore the microbial co-occurrence network and silage fermentation of Gliricidia and Leucaena prepared with Napier grass and corn stover. The findings suggested that a woody plant can be mixed with corn stover to make high-quality silage, which can alleviate the shortage of feed and promote local animal production. In another interesting investigation, Al-Askar et al. focussed on the bioprocessing of biomass residuals (peanut plant residual) into a beneficial substance (citric acid) using *Trichoderma longibrachiatum*. The crude citric acid that was obtained showed inhibitory potential against three toxinogenic fungi (*A. flavus, A. ochraceus*, and *F. oxysporum*) too. The study demonstrated the utility of T. longibrachiatum of an endophytic fungus to produce citric acid through the fermentation of peanut plant residual biomass. Native grass is widely utilized for grazing and haymaking, and is the prime source of forage in pastoral areas. Seasons affect the quality and productivity of native grass. Ensiling is a traditional method to preserve forage nutrients in the harvesting season. However, there is limited information available related to the microbial community and fermentation products during the ensiling process of native grass with additive treatments. Therefore, to improve the usability of native grass resources as feed, the effects of lactic acid bacteria and molasses additions on the microbial population, fermentation quality, and nutritional quality of native grass during silage were investigated by Li et al.. Outcomes of this study suggested that the supplementation of lactic acid bacteria and molasses improved the relative abundance of lactobacilli of native grass silage and enhanced the fermentation quality.

Daptomycin (a cyclic lipopeptide antibiotic) shows antibacterial activity against antibiotic-resistant Gram-positive bacteria. It is produced by *Streptomyces roseosporus via* non-ribosomal peptide synthetases. Lyu et al. successfully utilized multi-level metabolic engineering strategies in *S. roseosporus* to reconstruct high-quality daptomycin- overproducing strain L2797-VHb, including precursor engineering, regulatory pathway reconstruction, byproduct engineering, multicopy biosynthetic gene cluster, and fermentation process engineering.

The quality of cigar tobacco leaves is affected by the microbiota. Zheng et al. improved the quality of cigar tobacco leaves by fermenting it with *Acinetobacter* sp. 1H8 and *Acinetobacter indicus* 3B2. The inoculation of these two bacterial strains completely changed the original bacterial community. The study indicated the improvement of fermentation product quality by regulating microbial community, and gain insight into the microbial ecosystem.

Similarly Feng et al. studied the effect of exogenous electrons on electroactive *Escherichia Coli*, Shi et al. studied the catotenoid synthesis in *Phaffia rhodozyma*, Malla et al. used functional metagenomis approach for the identification of a novel efficient L-Lysine exporter. The production and characterization of lanthomicins by promoter engineering in *Streptomyces chattanoogensis* L10 was performed by Liu et al. Finally She et al. studied the yield improvements of Albofungins, and alpha amylase production using fermentation technology was researched by Geissler et al..

In summary, the articles published in the Research Topic “*Trends in applications and improved production of biologically active metabolites using microbial fermentations*” provides great insights on the novel approaches implemented (statistical optimization, metagenomics, metabolic engineering, whole genome sequencing, proteomics, and transcriptomic) not only in the quality improvement of various biomasses but also the enhanced production of biologically active metabolites from different microbes *via* fermentation process.

## Author contributions

All authors listed have made a substantial, direct, and intellectual contribution to the work and approved it for publication.

## Conflict of interest

The authors declare that the research was conducted in the absence of any commercial or financial relationships that could be construed as a potential conflict of interest.

## Publisher's note

All claims expressed in this article are solely those of the authors and do not necessarily represent those of their affiliated organizations, or those of the publisher, the editors and the reviewers. Any product that may be evaluated in this article, or claim that may be made by its manufacturer, is not guaranteed or endorsed by the publisher.
